# Surgical mentorship in low‐resource environments: Opportunities and challenges, a perspective

**DOI:** 10.1002/hsr2.2258

**Published:** 2024-07-30

**Authors:** Wireko A. Awuah, Joecelyn K. Tan, Hareesha R. Bharadwaj, Nicholas Aderinto, Tomas Ferreira, Heli Patel, Muhammad H. Shah, Abhay A. Kapoor, Sumitaksha Banerjee, Toufik Abdul‐Rahman, Oday Atallah

**Affiliations:** ^1^ Faculty of Medicine Sumy State University Sumy Ukraine; ^2^ Faculty of Medicine University of St Andrews St. Andrews UK; ^3^ Faculty of Biology Medicine and Health The University of Manchester Manchester UK; ^4^ Internal Medicine Department LAUTECH Teaching Hospital Ogbomoso Nigeria; ^5^ School of Clinical Medicine University of Cambridge Cambridge UK; ^6^ Faculty of Medicine Nova Southeastern University Dr Kiran C Patel College of Allopathic Medicine Davie Florida USA; ^7^ School of Medicine Queen's University Belfast Belfast UK; ^8^ Internal Medicine Department B.J. Medical College Ahmedabad India; ^9^ Department of Medicine Burdwan Medical College and Hospital Barddhaman West Bengal India; ^10^ Department of Neurosurgery Hannover Medical School Hannover Germany

**Keywords:** global surgery, low‐resource settings, surgery mentoring

## Abstract

**Background and aims:**

In low‐ and middle‐income countries (LMICs), a shortage of skilled surgical practitioners hampers healthcare delivery, impacting well‐being and economic growth. Surgical mentorship programs offer a promising solution but face challenges in implementation. This review aims to comprehensively assess the impact of surgical mentorship programs in LMICs and identify challenges and opportunities for their development and implementation.

**Methods:**

A thorough literature search was conducted from 2000 to 2023 using multiple databases, focusing on surgical mentorship programs in LMICs. Inclusion criteria encompassed full‐text articles in English that demonstrated characteristics of mentorship. Rigorous exclusion criteria were applied to ensure high‐quality evidence inclusion.

**Results:**

Surgical mentorship programs in LMICs strengthen local surgical capacity, improve surgical skills and patient outcomes, optimize resources and technology utilization, and positively impact medical students aspiring to be surgeons. However, challenges such as resistance to change, resource limitations, financial constraints, logistical and technological challenges, and time constraints hinder their implementation.

**Conclusion:**

Despite challenges, surgical mentorship programs hold promise for enhancing surgical capacity and healthcare quality in LMICs. Standardized metrics for accountability, innovative funding mechanisms, collaborative partnerships for scalability, interdisciplinary integration, and leveraging virtual mentorship programs are key strategies to overcome challenges and foster sustainable learning cultures, ultimately contributing to improved healthcare equity and quality in low‐resource settings.

## 1 INTRODUCTION

In low‐ and middle‐income countries (LMICs), a scarcity of skilled practitioners in surgical, obstetric, and anesthesia constitutes a significant impediment to healthcare delivery, predominantly stemming from inadequate local training infrastructures.^1^ This shortfall in skilled healthcare personnel has far‐reaching implications for the overall well‐being, socioeconomic development, and healthcare outcomes of these nations. The Lancet Commission on Global Surgery has aptly emphasized the imperative to nurture a robust surgical workforce as a critical component of the global health and development agenda.^1^ In LMICs, there is a severe shortage of specialized healthcare providers in the field of surgery as ascertained by the surgeon:patent ratio compiled by the aforementioned commission. These ratios for specialist surgical workforce (per 100,000 people) can be as low as 3:100,000 in South Asian countries like Bangladesh and Sri Lanka or 1:100,000 in African states like Nigeria and Uganda.^1^ This shortage creates a critical problem in the provision of surgical care. For example, nine out of every 10 individuals in LMICs lack access to basic surgical care.^1^ Additionally, LMICs, which account for 48% of the global population, have a disproportionately small workforce, where only 20% of all surgeons are observed.^2^ Consequently, there is a pressing need to increase the number of surgeons in LMICs to tackle the significant gap and improve access to surgical care; the target is to have 20 surgeons per 100,000 people by 2030.^3^

Surgical mentorship programs have emerged as a vital tool for strengthening surgical systems in LMICs.^4^ Mentorship, in this context, refers to a dynamic process where experienced and empathetic individuals, who are proficient in their respective fields, guide and coach others, either in person or virtually.^5^ This guidance aims to ensure proficient workplace performance and ongoing professional growth among surgical providers.^5^ Unlike traditional training approaches, which may struggle with transferring tacit knowledge and applied skills effectively, mentorship initiatives bridge the gap between initial training and the practical application of surgical expertise.^6^

Mentorship programs in LMICS operate through a multifaceted approach involving guided observation, personalized instruction, and context‐specific interventions.^6^, ^7^, ^8^, ^9^, ^10^ These interventions are tailored to meet the unique demands of the local healthcare landscape, which may include resource constraints, cultural consideration, and healthcare delivery challenges. There have been successful surgical mentorship initiatives that have resulted in some positive outcomes. Various mentorship programs in LMICs in Africa, Asia, and the Americas have all instilled confidence in surgical mentees, improved job satisfaction, and resulted in safer surgical procedures with favorable clinical outcomes.^7^, ^8^, ^9^, ^10^ By engaging in mentorship programs, surgical trainees not only develop technical skills but also gain insights into navigating the complexities of healthcare delivery in resource‐limited settings.

However, the execution of surgical mentorship initiatives in low‐resource settings comes with several challenges. Resistance to transformative changes within well‐established healthcare systems, compounded by the scarceness of healthcare providers, presents substantial barriers to the integration of these mentorship models.^11^, ^12^, ^13^, ^14^ Furthermore, concerns regarding the optimal frequency and duration of mentorship interactions, as well as the logistical complications inherent in their execution, increase the complexity to their seamless incorporation into existing frameworks.^9^, ^15^, ^16^, ^17^

This study presents a concise yet comprehensive overview of the transformative potential of surgical mentorship programs in improving surgical capability and healthcare quality in low‐resource environments such as LMICs and the challenges and opportunities inherent in their development and implementation.

## 2 METHODOLOGY

This review comprehensively assesses surgical mentorship programs in LMICs. The inclusion criteria for this review were limited to full‐text articles written in English, covering the period from 2000 to 2023. This timeframe was selected to facilitate a thorough evaluation of established practices within the field and to capture any significant advancements over a substantial period.

To ensure clarity regarding the inclusion of mentorship programs, the criteria encompassed not only explicitly labeled mentorship programs, but also training programs that demonstrated characteristics of mentorship, such as personalized guidance and ongoing professional development. To ensure an exhaustive literature search, several databases were utilized, including PubMed, EMBASE, Google Scholar, the Cochrane Library, and Scopus. The search terms employed included “surgical mentorship,” “medical education,” “LMICs,” along with additional terms such as “surgical training,” “clinical education,” and “professional development.”

In addition to database searches, additional sources were identified through a manual search of references cited in recent reviews focused on surgical education and training in LMICs. Rigorous exclusion criteria were applied, which involved excluding standalone abstracts, case reports, posters, and unpublished or non‐peer‐reviewed studies. These criteria were implemented to ensure the inclusion of high‐quality and reliable evidence.

The scope of the review did not impose a predetermined limit on the number of studies to be included, aiming to gather comprehensive knowledge of the subject matter. Various study designs were encompassed, including descriptive studies, cohort studies, and observational studies. The review also covered investigations conducted in both academic and clinical settings, providing a broad perspective on the use of surgical mentorship programs in LMICs.

The distinction between mentorship and conventional training approaches was addressed by focusing on the process rather than solely on the outcome. This approach aimed to highlight the unique characteristics of mentorship, such as personalized guidance and ongoing professional development, compared to more traditional training methods. A summary of the methodology employed is presented in Table 1.

**Table 1:** Summary of methodology for this review.

Methodology steps	Description
Literature search	PubMed, EMBASE, Google Scholar, the Cochrane Library, and Scopus
Inclusion criteria	− Full‐text articles published in English − Focus on surgical mentorship programs in LMICs − Adult and pediatric populations
Exclusion criteria	− Stand‐alone abstracts − Case reports − Posters − Unpublished or non‐peer‐reviewed studies
Search terms	Keywords such as “surgical mentorship”, “medical education”, “LMICs” coupled with indicators like “surgical training”, “clinical education”, and “professional development”
Additional search	− Manual examination of references cited in recent reviews focused on surgical education and training in LMICs − No predetermined limit on the number of studies − Encompassing diverse study designs: Descriptive studies Cohort studies Observational studies − Including investigations in both academic and clinical settings

Abbreviation: LMICs, low‐ and middle‐income countries.

## 3 TRANSFORMING SURGICAL LANDSCAPES: THE IMPACT OF SURGICAL MENTORSHIP PROGRAMS IN RESOURCE‐LIMITED SETTINGS

### 3.1 Strengthened local surgical capacity and workforce

Surgical mentorship programs play a pivotal role in enhancing the local healthcare workforce, encompassing not only surgical techniques but also preoperative and postoperative care, patient management, and the integration of new technologies.^18^ Some mentorship programs exhibit a unique focus on sustainability by aiming to train the trainers, empowering local healthcare professionals to educate and mentor others, thereby enabling local healthcare professionals to educate and mentor subsequent generations. This fosters a sustainable cycle of skill dissemination and professional advancement.^19^

Further, participation in a global mentorship network empowers local healthcare workers to advocate for systemic changes at both institutional and governmental levels. These programs also extend beyond clinical skill development to cover facets of healthcare administration, data management, and operational logistics. This comprehensive approach strengthens the entire healthcare system, thereby improving the quality and efficiency of surgical care delivery.^18^, ^19^ Global networks offer additional value in the form of collaborative research prospects, potentially leading to peer‐reviewed publications and the development of innovative surgical methodologies.^7^ Moreover, mentorship initiatives can incorporate elements of mental health support, stress management, and coping strategies. These are particularly beneficial for healthcare professionals, particularly surgeons, faced with the considerable emotional distress of practicing in resource‐constrained environments.^9^

Collectively, these diverse benefits converge to improve patient care standards. A skilled and adequately prepared workforce, seamlessly integrated into a functional healthcare system, significantly increases the likelihood of successful surgical interventions and other medical services.

### 3.2 Improved surgical skills, techniques, and patient outcomes

Within low‐resource settings, mentorship initiatives have emerged as transformative tools, impacting both the surgical proficiency of mentees and patient outcomes. Research highlights the critical role of mentorship in facilitating the transfer of tacit surgical knowledge and techniques, emphasizing the use of direct observation, coaching, and the provision of context‐specific practical solutions.^19^ These programs adopt a low‐dose, high‐frequency approach, whereby learning occurs in spaced intervals, aligning seamlessly with the preferences of surgical providers in resource‐constrained settings, thus fostering continuous growth.^19^

In parallel, collaborative models that leverage the synergetic amalgamation of personnel and resources offer a viable strategy to enhance surgical services, particularly in settings experiencing low patient volumes.^10^ In line with this, efforts to establish epilepsy surgery programs in resource‐limited settings provide more insight. For instance, a study conducted in Ho Chi Minh City, Vietnam, aimed to determine the outcome of such a program.^8^ The results indicated that out of 35 patients, 29 (82.8%) achieved Engel Class I outcomes, meaning they were free of disabling seizures, while 6 (17.2%) achieved Class II outcomes, indicating rare disabling seizures (“almost seizure‐free”).^8^ These findings underscore the potential impact of establishing and expanding epilepsy surgery programs in resource‐limited settings to improve patient outcomes and address the unmet needs of individuals with drug‐resistant epilepsy.

Skill retention, an essential metric for evaluating mentorship efficacy, has been demonstrated in several studies. For instance, telementoring initiatives in Mozambique have delivered measurable results, with mentees exhibiting consistently increased confidence and proficiency in advanced laparoscopic procedures for periods exceeding 1 year.^20^ Mentorship also broadens its scope to encompass nontechnical skills, including effective communication and teamwork, as evidenced in Tanzanian contexts.^15^ Here, the multicomponent mentorship program demonstrated significant improvements in safety practices by an additional 20.5% along with teamwork and communication conversations by 33.3%.^21^ Furthermore, for patient outcomes, the aforementioned study showcased that documentation of sepsis and surgical site infection diagnosis in patient files improved by 41.8% and 22.3%, respectively, and maternal sepsis rates reduced by 1.0% following intervention.^21^ Consequently, the impact of mentorship programs on patient outcomes is considerable. Studies from the African continent corroborate the positive influences of these programs, manifesting in improved management of infectious diseases and childhood maladies and improved laboratory services.^6^, ^21^ Such initiatives yield direct, measurable patient benefits: reduced postsurgical infection rates, the implementation of rigorous safety protocols, improved provider–patient communication, expedited patient recovery, and discernible reductions in decision‐to‐incision times.

### 3.3 Resource optimization and technology utilization

Surgical mentorship initiatives have exhibited proficiency in deploying innovative strategies focused on resource optimization. Such programs frequently commence with exhaustive workshops involving key stakeholders, ranging from healthcare administrators to clinicians. These workshops serve as platforms for collective problem‐solving, often employing visual methodologies like causal loop diagrams to identify the variables impacting resource use.^19^

Some initiatives also leverage mathematical modeling to forecast resource needs with precision.^19^ Using a comprehensive data set—encompassing costs from patient referrals to surgical procedures and the overheads of the mentorship program—these models enable a robust, quantitative assessment of return on investment. However, the evaluation metrics are not solely confined to quantitative analyses. Qualitative measures, such as skill acquisition and staff confidence, are equally emphasized, thereby adopting a more holistic strategy towards resource optimization.^10^ This dual focus enables the identification of both cost‐saving avenues, such as minimizing unnecessary patient referrals, and qualitative improvements in professional development.

One compelling example of this approach is the ‘surgathon’ model. This initiative unites students and faculty across disciplines to collaboratively address surgical challenges.^22^ Through a structured 10‐step plan, the program leverages existing resources, such as surgical instruments and healthcare personnel. Judging criteria emphasize impact, sustainability, and feasibility, thus fostering a culture of resource efficiency.^22^ Collaboration with external experts enriches the resource pool, leading to innovative solutions and the establishment of dedicated centers for surgical innovation.^23^

Furthermore, telemedicine has revolutionized the mentorship landscape by overcoming geographical constraints and enhancing real‐time guidance during surgical procedures.^7^, ^24^ Multiple studies have highlighted the dynamic learning environment facilitated by remote observation and guidance, enriching the mentorship experience by replicating the physical presence of mentors in operating rooms.^24^

Telemedicine serves as a critical means for instant problem‐solving, particularly in remote settings. Surgeons can consult experienced mentors in real time, thereby significantly improving patient outcomes.^7^, ^25^ In conjunction with other technology‐assisted mentoring tools, telemedicine provides mentees with an extensive array of educational resources, fostering interactive learning experiences.^20^

It is noteworthy that telemedicine, while facilitating mentoring interactions, yields broader and enduring effects beyond immediate mentoring sessions. The lasting influence of telemedicine on mentoring programs encompasses enhancements in surgical outcomes, augmented accessibility to specialized expertise in remote locales, and the formulation of culturally sensitive mentoring endeavors.^7^, ^25^ Although initial efforts have laid the groundwork for culturally attuned programs, persistent challenges remain in ensuring equitable access and mitigating linguistic and cultural impediments.^26^

### 3.4 Positive impacts on medical students aspiring to be surgeons

In low‐resource settings, the impact of surgical mentorship programs on medical students aiming for a surgical career is significant. These programs connect aspirants with experienced mentors, offering both theoretical and practical expertise gained through years of surgical practice.^27^ Such initiatives extend beyond traditional education by nurturing skills and fostering early interest in surgical specialties.^27^

Similarly, these initiatives often provide students with opportunities for professional networking by connecting them with mentors for career development, research opportunities, and residency placements.^21^ This exposure instills knowledge, resilience, adaptability, and problem‐solving skills. It transforms them into adaptable professionals ready to tackle the multifaceted complexities of surgical practice in their immediate surroundings. Moreover, hands‐on clinical experience is integral to becoming a surgeon.^28^ Many mentorship programs seamlessly integrate this component, allowing students to actively engage in surgical procedures under the vigilant guidance of experienced surgeons.^21^ This practical exposure bridges the gap between theory and practice, nurturing confidence and interest.

Beyond the confines of the operating room, some mentorship programs foster research and innovation.^4^, ^11^ They inspire students to explore novel solutions to the healthcare challenges endemic to low‐resource settings. This exposure nurtures the art of critical thinking and instills a deep commitment to advancing surgical care in resource‐constrained environments.

## 4 CHALLENGES IN IMPLEMENTING SURGICAL MENTORSHIP INITIATIVES IN RESOURCE‐LIMITED SETTINGS

Surgical mentorship programs present a promising avenue for improving surgical care in low‐resource settings. However, their widespread implementation is not without challenges. Understanding these challenges is critical for developing targeted interventions that can facilitate the adoption of such programs.

### 4.1 Resistance to change

The effectiveness of global mentorship programs is dependent on a positive perception of their inherent value.^11^ Despite studies highlighting the benefits of such programs, their full implementation in low‐resource settings often encounters resistance to change.^12^, ^13^ This resistance can manifest as hesitancy among participants to integrate newly acquired skills into their surgical practices.^14^ Various factors contribute to this resistance, such as mentee–mentor dynamics, levels of mentee engagement, and mentor availability.^14^, ^29^ For example, it was discovered in a study conducted during the SS2020 initiative in Tigray, Ethiopia, which aimed to improve access to safe, high‐quality surgical care in resource‐constrained rural hospitals, that interactions between mentees and mentors were critical to the mentorship program's success.^7^ However, due to perceived differences in expertise and communication styles, some mentees needed to be more open to connecting with their mentors fully. The SS2020 program organizers created a structured feedback method to address this difficulty and foster more effective mentee–mentor relationships.

In addition to considering the dynamics between mentees and mentors, it's crucial to recognize that resistance to change can vary significantly among different cadres of healthcare providers and across various healthcare facility sizes. Senior or high cadre surgical providers, often possessing extensive experience and entrenched practices, may exhibit greater resistance to adopting new techniques or methodologies compared to their junior or low cadre counterparts. This resistance may stem from a reluctance to depart from established practices, concerns about the feasibility or efficacy of new approaches, or simply a preference for the familiar. Moreover, differences in resistance to change can also be observed across healthcare facility sizes. Small health facilities, such as rural clinics or district hospitals, often operate with limited resources, infrastructure, and staffing levels.^30^ In such settings, the introduction of these new initiatives may encounter resistance due to concerns about feasibility, cost‐effectiveness, or compatibility with existing workflows.^30^ Additionally, smaller facilities may face logistical challenges in implementing and sustaining change initiatives, such as training staff, acquiring necessary equipment, or establishing supportive policies and procedures.

### 4.2 Resource limitations

In low‐resource settings, the scarcity of experienced surgical mentors presents a critical challenge that directly affects the availability of mentoring for trainees, thus compromising the quality of mentorship.^9^ For example, due to a scarcity of experienced surgical mentors, several Anglophone Caribbean nations’ training of surgical mentees on how to use minimally invasive surgical procedures have been hampered.^9^ In addition, there is often an inadequacy in the scope and depth of surgical education and training within these regions.^9^ This shortcoming adversely affects not only trainees but also mentors, who may lack access to the latest surgical techniques and guidelines. The situation is further exacerbated by the lack of specialized surgical facilities and equipment.^15^ The absence of adequately equipped operating theaters and sterile environments impedes effective mentorship, constituting a challenge for both mentors and mentees.^15^

Global mentorship programs face substantive barriers due to inherent resource limitations, particularly in settings already characterized by scarcity. The availability of qualified personnel for mentorship roles is often hindered by the overwhelming workload within these environments. Alidina and colleagues’ research in Tanzania highlights the logistical difficulties encountered by surgeons, which are magnified by an existing dearth of surgical expertise.^7^ This leads to mentors having inadequate time to effectively engage in educational endeavors. Parallel findings are reported in Ethiopian studies, illustrating the challenge mentors face in dedicating adequate time to mentees owing to significant professional commitments.^21^ Such conditions may lead to reduced mentor–mentee interaction, undermining the efficacy of mentorship initiatives. It further contributes to a cycle of reduced participation, limiting the intended impact of mentorship programs.^9^, ^18^

### 4.3 Financial constraints and compensation dilemmas

Sustainable operation of surgical mentorship programs requires considerable financial investments for mentor recruitment, mentorship training, and ongoing operational support.^16^ In low‐resource settings, securing and maintaining such resources can be a significant financial burden.^21^ Suboptimal remuneration of mentors exacerbates this challenge, leading to reluctance among experienced professionals to contribute their expertise to such programs.^29^ Additionally, departmental leaders’ hesitance to increase budgetary allocations further compounds this issue.^18^, ^29^ Such hesitance often originates from either competing departmental priorities or an insufficient appreciation of the long‐term benefits of mentorship programs.^29^ In these situations, organizations may explore external sources of funding, such as philanthropic contributions or industrial partnerships. However, securing such funding is not only difficult but also introduces ethical complexities related to potential conflicts of interest.^16^

### 4.4 Logistical and technological challenges

Logistical complexities present additional barriers to the effective implementation of surgical mentorship programs. Modern approaches to surgical mentorship increasingly leverage telemedicine and digital tools for remote guidance and training.^10^ Yet, in resource‐limited settings, unreliable internet connectivity and access to advanced telecommunication systems undermine the efficiency of tele‐mentorship efforts.^31^ Telementorship programs in Ecuador, for example, found challenges with internet connectivity and inconsistent phone services.^31^ Furthermore, mentorship programs in Tanzania encountered logistical challenges such as issues with the timing of mentorship visits, conflicting schedules of telementoring sessions with their regular working hours, and language barriers when working with an international faculty.^21^ The requirement for physical interaction in some instances further exacerbates these challenges. The need for travel to remote locations—areas often with inadequate transport infrastructure—impedes the ability of mentors to provide timely and effective support.^32^ These geographical limitations add an additional layer of complexity to logistical planning.^10^, ^17^, ^32^

Furthermore, language barriers also present a difficulty in the execution of surgical mentorship programs. This is exemplified by research in the Democratic Republic of the Congo.^18^ The confluence of inadequate transport facilities, suboptimal infrastructure, and safety concerns compounds the logistical and technological hindrances encountered during mentorship programs.^18^, ^21^

### 4.5 Time constraints

Time constraints often present a significant challenge in the optimization of surgical delivery via mentorship programs in resource‐deprived settings. These limitations arise at various stages within the mentorship framework and have a considerable impact on overall program efficacy. A pivotal factor contributing to time constraints is the limited availability of experienced mentors. Skilled mentors are often in high demand globally, dedicating their time to clinical practice, teaching, and research.^33^ This situation results in extended waiting periods for prospective mentees and frequently reduces the duration and frequency of interactions between mentors and mentees. The resultant curtailed mentorship durations compromise the depth of the mentor–mentee relationship and generally offer restricted avenues for mentees to acquire comprehensive practical experience.^7^ Thus, the pressure to navigate time constraints typically requires a compromise between mentorship quality and operational efficiency. Balancing these competing demands frequently emerges as a delicate challenge. Survey responses from a study conducted in five rural Ethiopian facilities after a surgical mentorship program shed light on these challenges. Mentees frequently cited issues related to the duration of mentorship visits, unforeseen emergencies, and fluctuations in surgical caseloads, all of which constrained the availability of time for hands‐on surgical mentorship.^7^ These findings underscore the pervasive nature of time constraints in mentorship programs, highlighting the need for innovative solutions to address this critical aspect of surgical capacity building in resource‐limited settings.^7^

## 5 PIONEERING THE PATH AHEAD: FUTURE PROSPECTS FOR SURGICAL MENTORSHIP IN LOW‐RESOURCE SETTINGS

The prospective impact of surgical mentorship programs in low‐resource settings has promising potential for transformative advancements. As the landscape of global surgical practice evolves, strategic planning and policy considerations become integral to reinforcing such initiatives.

### 5.1 Standardizing metrics for accountability and improvement

The development of standardized metrics for accountability and improvement is important for the ongoing viability and effectiveness of surgical mentorship initiatives in low‐resource settings.^7^ Such metrics foster a culture committed to accountability, transparency, and evidence‐based decision‐making within the mentorship framework.^33^ By holding all involved parties—from program coordinators to mentors and mentees—accountable, these metrics provide a robust mechanism for oversight.^33^ Importantly, they equip policymakers with empirically grounded information, enabling targeted assessments of mentorship scheme effectiveness and resource distribution, particularly important in low‐resource settings.^33^ Furthermore, clear, quantifiable outcomes are vital for the appraisal of mentorship initiative.^7^ Metrics such as enrollment figures, the number of active mentor–mentee relationships, the frequency and duration of mentoring sessions, and actionable insights gleaned or notes recorded can serve as invaluable indicators of program efficacy.^7^ These metrics not only offer a quantitative assessment of impact but also provide insights into the initiative's reach, level of engagement, and overall effectiveness. However, while implementing a policy to establish standardized accountability metrics, policymakers must exercise cultural sensitivity. The chosen metrics should consider the local customs and practices prevalent in LMICs, which can pose challenges when incorporating them into standardized frameworks. Therefore, it becomes imperative to engage in cultural adaptation, involving efforts to comprehend the cultural nuances and adjusting metrics accordingly. Achieving this necessitates active engagement with local communities and healthcare providers. It is crucial to involve all pertinent stakeholders, including healthcare providers, policymakers, and patients, in developing metrics to ensure acceptance and eventual success.

### 5.2 Innovative funding and support mechanisms

Securing stable funding mechanisms constitutes a critical facet for the future sustainability of surgical mentorship programs in low‐resource settings.^34^ Policymakers face the complex task of identifying consistent funding sources beyond traditional avenues. The exploration of alternative funding streams, such as crowdfunding and public–private partnerships, warrants consideration.^35^ Governmental grants and financial incentives could offer reliable support, particularly if administrative processes are streamlined to encourage private sector participation.^35^ Sustainable financial models, bolstered by stringent monitoring and evaluation protocols, contribute to long‐term program resilience while maintaining transparency and accountability.^34^ The incorporation of international collaboration further diversifies funding avenues and ensures continuous program backing.^34^ However, governmental agencies in LMICs may be more inclined to prioritize investment to reduce inequalities in other sectors such as energy, transport infrastructure, and early formal education. Providing transparent insights and plans concerning the allocation and utilization of the finances in implementing the mentorship programs may attract more partnerships with local and international public and private bodies to create a robust funding network. Policymakers could also host fundraising events such as charity galas and seek participation from medical students, residents, and academic institutions.

### 5.3 Enhancing collaborative partnerships for scalability

Expanding surgical mentorship initiatives into scalable programs requires a multifaceted approach that recognizes the pivotal role of policy changes. A significant challenge is navigating the complexities of policy changes to align with expanding mentorship programs. Policymakers face challenges in revising existing policies or crafting new ones that cater to the specific needs of scalable mentorship initiatives. These challenges include bureaucratic resistance and the alignment of policies among multiple stakeholders.

The expansion of surgical mentorship initiatives into scalable programs will require a structured strategy for improving multistakeholder collaboration. A comprehensive framework for resource optimization and collaboration across governmental bodies, nongovernmental organizations, academic institutions, and healthcare organizations is imperative.^36^ Policies and partnership agreements must be devised to facilitate equitable resource distribution while safeguarding the rights and interests of all contributors.^36^ Technological solutions, including telemedicine and virtual platforms, can improve knowledge dissemination and mentorship engagements, particularly in geographically isolated regions.^32^ Initiatives focusing on cross‐cultural training and sensitivity are essential for fostering effective stakeholder communication and cooperation.^33^

### 5.4 Interdisciplinary integration for comprehensive care

Incorporating an interdisciplinary methodology into surgical mentorship programs offers a nuanced strategy for addressing the complex healthcare demands of low‐resource populations. Policies should incentivize collaboration among various medical disciplines by facilitating cross‐disciplinary dialogues and supporting joint training programs.^37^ International partnerships with institutions and experts from different countries can provide mentorship programs with diverse perspectives and experiences.^37^ Similarly, policies should establish platforms and forums where professionals from various surgical fields can freely exchange ideas, experiences, and best practices, fostering a culture of interdisciplinary cooperation for effective surgical mentorship programs. Joint training programs that involve multiple surgical specialties can be complex to organize due to varying coordinating schedules and the curriculum development of different faculties and hospitals. Additionally, aligning educational goals and objectives across other specialties can be difficult. As such, the relevant stakeholders must provide vital support by defining program objectives, allocating resources, and creating a regulatory framework that guides curriculum development.

### 5.5 Leveraging virtual mentorship programs

Virtual mentorship schemes signify a paradigm shift in surgical education and knowledge dissemination. These programs are particularly advantageous in remote or low‐resource settings where physical mentor access is constrained.^38^ For example, the use of a telecollaboration‐mentorship strategy in Sri Lanka, Bangladesh, and India has demonstrated its viability, sustainability, scalability, and reproducibility in expanding access to epilepsy surgery in LMICs. This strategy has the potential to improve surgical candidacy selection, increase the variety of accessible surgical procedures, and pave the way for the construction and operation of new epilepsy surgery centers in LMICs.^17^ To successfully expand these telementoring ideas in other LMICs, policies should target the inclusion of the assurance of digital equity, encompassing improved internet connectivity, equitable device accessibility, and digital literacy programs.^39^ Also, robust regulatory infrastructure is necessary to maintain security and confidentiality in virtual mentorship programs to deal with ethical concerns such as data mishandling, breaches, and unauthorized access. Effectively, policies should outline stringent data protection measures, privacy standards, and mechanisms to secure virtual interactions, instilling confidence in participants. Specialized preparatory training for mentors and mentees engaged in virtual mentorship is indispensable, and international collaborations should be encouraged to enrich these programs and foster a globally interconnected learning community.

## 6 CONCLUSION

In summary, surgical mentorship programs have gained prominence as a viable avenue for addressing the pressing challenges in surgical care delivery in LMICs. Although these programs hold considerable promise for bridging the gap between theoretical knowledge and practical skills, there are several challenges to their effective implementation. These include resistance to organizational change, resource limitations, financial constraints, logistical complexities, and technological disparities. Nevertheless, the adoption of standardized evaluative metrics and innovative funding models, the promotion of interdisciplinary alliances, and the use of virtual platforms can collectively enhance surgical capacity, foster a sustained learning culture, and ultimately contribute to improved healthcare equity and quality in low‐resource settings.

## AUTHOR CONTRIBUTIONS

The study was conceptualized and supervised by Wireko A. Awuah. Material preparation, data collection, and analysis were performed by all authors. The first and final draft of the manuscript was written by all authors. All authors have read and approved the final version of the manuscript. Wireko A. Awuah had full access to all of the data in this study and takes complete responsibility for the integrity of the data and the accuracy of the data analysis.

## CONFLICT OF INTEREST STATEMENT

The authors declare no conflict of interest.

## ETHICS STATEMENT

Not applicable.

## TRANSPARENCY STATEMENT

The lead author Wireko A. Awuah affirms that this manuscript is an honest, accurate, and transparent account of the study being reported; that no important aspects of the study have been omitted; and that any discrepancies from the study as planned (and, if relevant, registered) have been explained.

## Data Availability

Data availability is not applicable to this article as no new data were created or analyzed in this study.
